# Using the Relative Energy Gradient Method with Interacting Quantum Atoms to Determine the Reaction Mechanism and Catalytic Effects in the Peptide Hydrolysis in HIV‐1 Protease

**DOI:** 10.1002/chem.201802035

**Published:** 2018-07-03

**Authors:** Joseph C. R. Thacker, Mark A. Vincent, Paul L. A. Popelier

**Affiliations:** ^1^ Manchester Institute of Biotechnology (MIB) 131 Princess Street Manchester M1 7DN UK; ^2^ School of Chemistry University of Manchester Oxford Road Manchester M13 9PL UK

**Keywords:** enzymes, peptides, quantum chemical topology, quantum atoms, reaction mechanisms

## Abstract

The reaction mechanism in an active site is of the utmost importance when trying to understand the role that an enzyme plays in biological processes. In a recently published paper [*Theor. Chem. Acc*. **2017**, *136*, 86], we formalised the Relative Energy Gradient (REG) method for automating an Interacting Quantum Atoms (IQA) analysis. Here, the REG method is utilised to determine the mechanism of peptide hydrolysis in the aspartic active site of the enzyme HIV‐1 Protease. Using the REG method along with the IQA approach we determine the mechanism of peptide hydrolysis without employing any arbitrary parameters and with remarkable ease (albeit at large computational cost: the system contains 133 atoms, which means that there are 17 689 individual IQA terms to be calculated). When REG and IQA work together it is possible to determine a reaction mechanism at atomistic resolution from data directly derived from quantum calculations, without arbitrary parameters. Moreover, the mechanism determined by this novel method gives concrete insight into how the active site residues catalyse peptide hydrolysis.

## Introduction

The quantum physics governing a chemical system is complex: each atom interacts with every other, according to various energy types: electrostatic or Coulomb, exchange and electron correlation. Moreover, from a physical point of view, kinetic energy also plays a role although this role is rarely discussed in chemistry textbooks, if at all. Despite this underlying physical complexity, a chemist routinely singles out a few atoms in a given system in order for them to explain the behaviour of the total system. For example, textbooks will tell that the base‐pair complex guanine⋅⋅⋅cytosine is more stable than the adenine⋅⋅⋅thymine complex because the former has three hydrogen bonds while the latter has only two. However, it was shown that this explanation is not correct in work^1^ that introduced the so‐called secondary interaction hypothesis but later it was shown^2^, ^3^ that this hypothesis is not correct either. This situation proves the need for a modern and rigorous protocol to bridge the gap between quantum mechanical data and a chemical rationale. In summary, the main question one faces is which fragment of a total system dictates the behaviour of the total system, in terms of energy profiles. More precisely, two questions can be posed: “which atoms are involved in determining the behaviour of a system?” and “which energy types determine the total behaviour of the system?”

In a recently published paper^4^ we proposed an answer to these two questions by introducing the so‐called Relative Energy Gradient (REG) method. The REG method is an exhaustive, parameterless and general method that can be applied to any energetically partitioned potential energy surface (PES). In essence, the REG method enables (i) the determination of a subset of partitioned energies that best describe the total behaviour of the system, and (ii) the extraction of chemical insight from an energetically partitioned system. The REG method was designed to allow for the automated analysis of arbitrarily sized chemical systems, and is particularly useful for large systems. Indeed, as the system size increases, the number of energy terms increases dramatically, making it unwieldy and even impossible to study large systems manually.

In our original paper^4^ we showed how the REG method detected the energy terms that most determine the behaviour of the water dimer as the distance between the two monomers varied. It was shown that the energy term that most favours binding in the water dimer is the classical (electrostatic) term between the hydrogen bond donor and the hydrogen bond acceptor atoms. The purpose of the current paper is to show that the REG method is a general and powerful tool that not only offers insight into the local stability preference in small molecules (e.g. the gauche effect^5^) but also elucidates chemical mechanism in biomolecular active sites. In the latter, the REG method operates on an overwhelmingly large amount of data. We show that the REG method can be used to pinpoint atoms and functional groups that facilitate and inhibit reactions in a protein's active site.

In this study, the so‐called Interacting Quantum Atoms (IQA) method is used in conjunction with the REG method. The IQA method was inspired by early work^6^ and is derived from the Quantum Theory of Atoms in Molecules (QTAIM),^7^, ^8^, ^9^, ^10^, ^11^ an established and popular^12^ electron density partitioning method. The domain of applicability of QTAIM is large and has also been used in QSAR studies.^13^, ^14^, ^15^ The IQA approach requires integrating over the electron density within QTAIM atoms, also known as topological atoms, in order to obtain well‐defined intra‐atomic and inter‐atomic energies.^16^, ^17^ The IQA approach has been used successfully in a number of papers, including (but not limited to) the following case studies: the nature of halogen interactions in perhalogenated ethanes,^18^ prototype S_N_2 reactions,^19^ proton‐transfer reactions,^20^ intramolecular bond paths between electronegative atoms,^21^ hydrogen‐hydrogen interactions with respect to the torsional barrier in biphenyl,^22^ short‐range electrostatic potentials across torsional barriers,^23^ CO_2_ trapping by adduct formation,^24^ atom‐atom repulsion as Buckingham potentials,^25^ and the diastereoselective allylation of aldehydes.^26^ All these studies focused on small molecules, whereas in this paper we show how IQA is able to give conclusive results when applied to large biomolecules by using it in conjunction with the REG method.

In principle, the REG method can be applied to any energy partitioning scheme but the IQA method offers a number of attractive features. In particular, when compared to other energy partitioning methods, such as Energy Decomposition Analysis (EDA)^27^, ^28^ for example, there are three reasons supporting the combination of REG with IQA.

The first is that IQA provides a definition of an atom, unlike EDA. The definition of an atom enables assigning energies to atoms and the pairwise interactions they are involved in. IQA provides chemically intuitive^29^ and meaningful energies. Indeed, the three major energy components of IQA ($E_{intra}^{A}$ , $V_{xc}^{AB}$ and $V_{cl}^{AB}$ ) respectively relate to an atom's internal energy, the covalent interaction energy between atoms A and B , and the “classical” Coulomb interaction energy between atoms A and B . These contributions can be treated on an equal footing within IQA. In contrast, EDA only partitions complexes into their monomeric constituents (or even molecular fragments), thus not offering information at atomic resolution. Moreover, EDA requires a reference state,^30^ whereas IQA does not.

A second reason for using IQA is the space‐filling nature of the topological atoms, which means that (i) there are no overlapping areas of electron density, and that (ii) there are no gaps between the atoms, that is, each point in space belongs to a topological atom. This space‐filling property prevents the over‐counting of electron density and also ensures that all electron density is accounted for (allowing for a very small loss due to a constant electron density envelope at the outer edge of a molecule). The latter property prevents under‐counting of electron density. The space‐filling nature of the topological atoms leads to the additivity of their properties. In other words, the energy of a functional group is simply the sum of the energies of the constituent atoms (with a small numerical error introduced by the integration method used).

A third and final reason is that IQA also has three advantages over Natural Bond Orbitals (NBO). First, IQA allows for well‐defined energy terms to be analyzed, whereas NBO only approximates the electrostatic energy within a molecular system. The reason for NBO's approximation is that the electrostatic interactions and “steric” interactions are convoluted within the so‐called Lewis‐type energy. This convolution is due to the use of a reference state within the method.^31^, ^32^ Secondly, IQA is able to describe “through‐space” interactions, whereas NBO is not.^33^ Thirdly, Stone^34^ showed that NBO is particularly prone to errors due to basis set superposition, while IQA is not.

In summary, we decided to move forward with IQA because of its atomic resolution, its independence of reference states, its full and clear separation of the different types of energy contribution, and its capacity to reveal “through‐space” interactions. However, we note that IQA is computationally expensive due to the finite volume and complicated shape of topological atoms.

It is possible to use alternative metrics to determine reaction mechanism. Such methods include the “Bond Evolution Theory”, in which the Electron Localisation Function is used to study the “flow of electron density” within a system.^35^ Another such example (involving electron density) has been shown to be a poor indicator of bonding is that of QTAIM′s bond critical points, which can vanish upon nuclear vibrations.^36^ By using the IQA method to determine the reaction mechanism we are using a physically robust methodology. This is because energy is a definite measure of relative stability and can therefore indicate bonds as energetically favourable exchange‐correlation energies between atoms.

**Figure 1:**
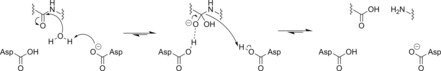
The traditional mechanism for the peptide hydrolysis reaction in aspartic proteases. The forward direction of this reaction occurs from left to right, a convention that is maintained throughout this article. The straight arrows represent the overriding direction from reactant (left) to products (right), over the transition state (middle).

Aspartic proteases are a common type of protease enzyme. Their active sites contain aspartate residues, which activate water to catalyse peptide hydrolysis. Figure 1 outlines the traditional mechanism^37^, ^38^ of peptide hydrolysis in aspartic proteases.

Peptide hydrolysis is an important step in the action of many diseases such as breast cancer, malaria, hypertension, Alzheimer's disease and the human immunodeficiency virus (HIV).^39^, ^40^, ^41^ Concerning the HIV, the aspartic protease site (alongside the reverse transcriptase^42^ and the integrase^43^ sites) is one of three active sites commonly used as drug targets. The protease site is a common drug target due to its important role in replicating the HIV.^44^ There has been much research into the inhibition of the aspartic protease active‐site^45^, ^46^ including research into dual‐acting drugs that, for example, inhibit multiple sites relevant in the replication of the HIV.^47^ The traditional mechanism, shown in Figure 1, consists of two main features. The first is nucleophilic attack of the water upon the peptide carbon atom to form the transition state. The second is the hydrolysis of the peptide bond in the transition state, in which the carboxyl groups of the aspartate play a crucial role. In this reaction the aspartate groups play a catalytic role, by hydrogen donation and extraction.

## System Details

**Figure 2:**
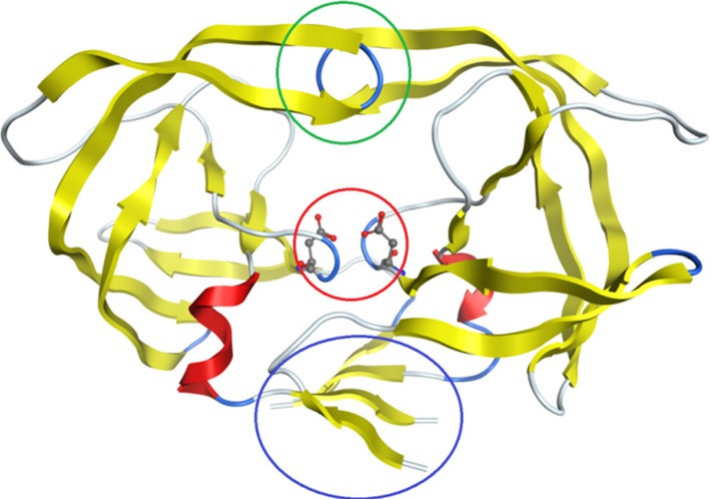
The overall tertiary structure (from crystallographic measurements) of the HIV‐1 Protease (PDB Code: 4HVP). This protein is made up of two subunits (one on the left and one on the right). The encircled regions highlight important features of the protein: flaps (green), aspartic groups in the active site (red), dimerisation domain (blue).

Figure 2 shows the overall tertiary structure, obtain from a crystallographic measurement, of the HIV‐1 Protease (PDB Code: 4HVP).^40^ This protein is made up of two subunits, one on the left and one on the right. Important regions of the protein consist of flaps, aspartic groups in the active site and the dimerisation domain. These regions are encircled in Figure 2. This protein is an enzyme that activates a water molecule, which then performs a nucleophilic attack on the carbonyl carbon of the scissile peptide bond, as explained below.

**Figure 3:**
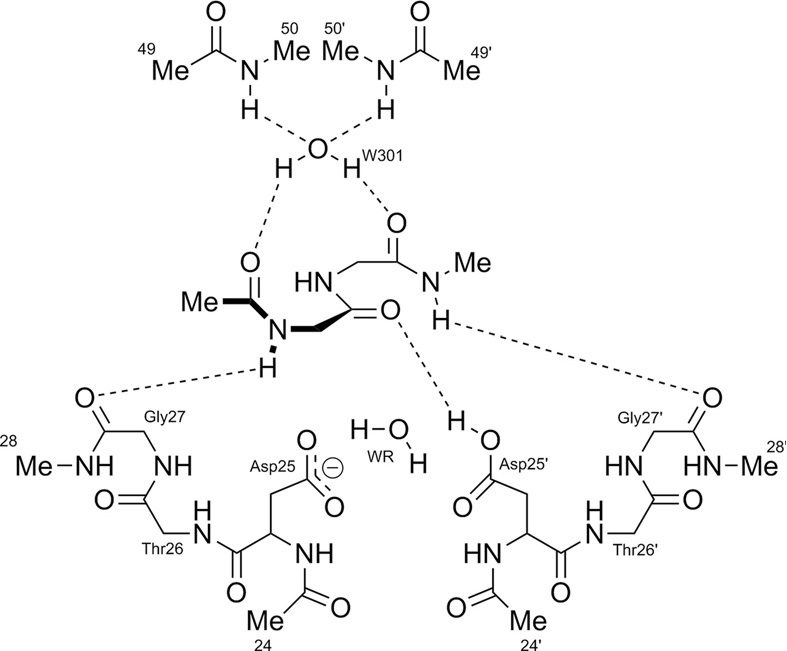
A schematic representation of the 133‐atom system obtained after truncating the HIV‐1 Protease 4HVP (Figure 2), which contains the active site studied. The system shown here corresponds to red‐encircled region in Figure 2 and the bottom part of the green‐encircled region. Hence, the residues 49, 49(′), 50 and 50(′) are representations of the flaps (green circle in Figure 2) while the rest represent the active site (red circle in Figure 2).

Computationally, it is not possible to handle the whole protein, that is, finding a SCF‐converged wave function within a reasonable time and perform an IQA analysis on it. Hence the protein was truncated to the same size as that in the study by Garrec et al.48a in 2011. Note that a more recent paper48b from a different group also studied this system but appeared after the current work had started. Garrec et al. give a detailed discussion as to why this is an appropriate model, which can perhaps be summarised as follows: the model is large enough to include all the important direct and indirect energetic interactions (vide infra), but at the same time it remains small enough to be described at a reasonable level of theory. Following their model, we terminate the residues with a methyl group attached to the peptide linkage and, in addition, the methyl groups of threonine residues are replaced by hydrogen. Figure 3 schematically shows this truncated system which contains 133 atoms (only a few hydrogen atoms are shown for clarity), and for which the wave function and the IQA analysis could be obtained. Thus, this model has each of the aspartate residues (Asp25 and Asp25(′)) bonded to two other amino acids, leading to two amino acid chains: Asp25‐Thr26‐Gly27 and Asp25(′)‐Thr26(′)‐Gly27(′). We shall refer to one chain as “primed” and the other as “unprimed”, which relates to the notation used in the text and figures. Such a chain of three amino acids is often referred to as a catalytic triad.^49^ The primed and unprimed catalytic triads have a rigid, coiled structure, such that they maintain close proximity. This rigidity is due to a number of hydrogen bonds that naturally occur in the enzyme and are present in our model and are referred to as the “fireman's grip”.^50^ Another reason for this rigidity, proposed by others, is that it is induced by the peptide dipole moments in Thr26(′)‐Gly27(′) that interact with the negatively charged Asp25(′) residues.^50^, ^51^, ^52^ This rigidity, which is due to the fireman's grip and peptide dipole moments, is crucial in keeping the Asp25 and Asp25(‘) carboxyl groups coplanar, which, in turn, is crucial for the active site to act as a catalyst. Hence we consider it crucial to include the catalytic triad in the wave function calculation in order to maintain the rigid structure and coplanarity required for catalytic behaviour.^53^, ^54^, ^55^ Furthermore, when choosing more atoms with which we represent the system, we considered that the inhibition of the active site by a substrate can cause changes in the tertiary structure of a protein. In a crystallographic study^40^ of HIV‐1 Protease it was found that the presence of a ligand bound in the active site caused changes as large as 7 Å in the backbone (a region of the often described as the “flaps”, see Figure 2). These large atomic displacements indicate that these “flaps” may be important in the catalytic behavior of the system. Therefore, the truncated system studied contains a representation of these “flaps” in the form of two doubly methylated peptide bonds (residues 49(′), 50(′), 49 and 50).

The two water molecules in Figure 3, the reactive water molecule WR and W301, were not present in the crystal structure 4HVP. Hence they had to be added to the crystal structure, which was carried out before by others.48a The WR water molecule is crucial as it is needed to act as the nucleophile in the peptide hydrolysis reaction. The two Me49(′)−Ile50(′) groups are bonded to the “flaps” (in Figure 2) and the role of the W301 water molecule is to maintain cohesion of these groups and that of the rest of the system. The NH groups in the Ile50/50(′) amino acids strongly bind the W301 water molecule, which in turn binds to the substrate and ensures that the substrate geometry is correct for peptide hydrolysis.

**Figure 4:**
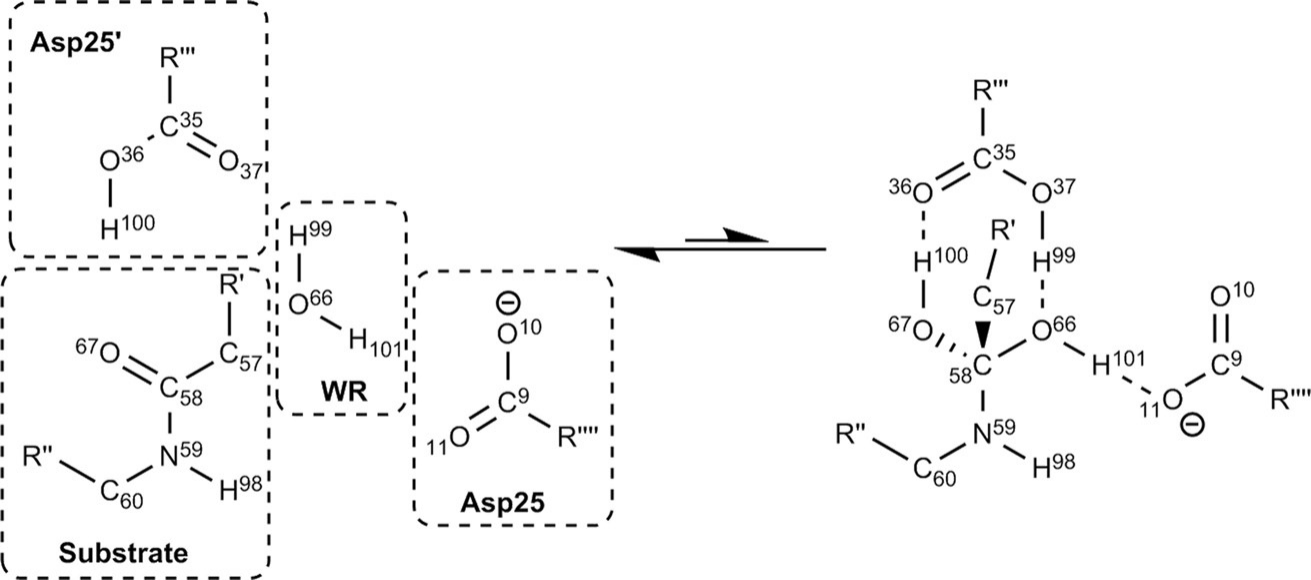
A small area of the active site studied (the central part in Figure 3), showing the atomic numbering used throughout this paper. Because they are the only groups required in the analysis, only the Asp25 and Asp25(′) carboxyl groups, the reactive (WR) water molecule, and the peptide area of the substrate are shown. The structure on the right corresponds to the transition state, and the straight reaction arrows reflect that the energy of this state is higher than that of the left structure. Note that the similar arrows in Figure 1 represent the overall direction from reactant to products, and hence face the opposite way.

The reaction‐step and atomic numbering used throughout this paper is shown in Figure 4. This region is highlighted as the atoms in this region are used throughout the Results and Discussion.

## Results and Discussion

**Figure 5:**
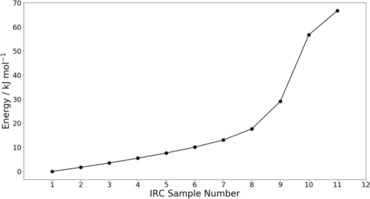
The energy profile of the reaction shown in Figure 4, which includes all 133 atoms shown in Figure 3. The energy at IRC sample number 1 corresponds to that of the reactants shown on the left in Figure 4 while the energy at IRC sample number 11 corresponds to the transition state shown on the right of Figure 4.

Figure 5 shows the total energy Potential Energy Surface (PES), starting from the reactants at Intrinsic Reaction Coordinate (IRC) sample number 1, and ending at the transition state at IRC sample number 11. This path covers a total energy change of 66.8 kJ mol^−1^. In this study we follow the IRC from left to right in Figure 5. Doing this gives information regarding the first step of the peptide hydrolysis, from which it is possible to determine the reaction mechanism using the REG method in conjunction with IQA. Calculation of the reaction mechanism will be guided by the REG method when applied to pairwise atomistic IQA energies. Firstly, we will evaluate the exchange‐correlation energies (commonly associated with covalency) to show an energetic reaction mechanism that is consistent with the traditional chemical interpretation of a reaction mechanism (i.e. covalent bonds forming and breaking). Secondly, we will discuss the electrostatic energies associated with the peptide hydrolysis, showing that the REG method is able to recover common chemical ideas (such as electrostatic hydrogen bonds and electrostatic nucleophilic attack) from physical principles. Finally, we will discuss the V_inter_(A,B) terms. These terms are the sum of pairwise electrostatic and exchange‐correlation energies and as such give a more complete overall picture of the reaction mechanism. Using V_inter_(A,B) terms allows for the assessment of cancellations in the exchange‐correlation and electrostatic energies, and returns total pairwise interaction energies between atoms.

### Determining the mechanism of peptide hydrolysis

To determine the mechanism of peptide hydrolysis in the HIV protease site, the exchange‐correlation IQA energies were evaluated using the REG method. The exchange‐correlation energy relates to the chemical concept of covalency, and thus to the making and breaking of bonds. This explains why we analyse this particular type of energy separately, in order to determine the reaction mechanism as traditionally thought of by chemists. The exchange‐correlation IQA energies were ordered (as discussed in the Theoretical and Computational Background), with the more negative REG values considered to be more important in defining the reaction mechanism than the less negative (or even positive) REG values are. This ordering from most negative to most positive is evident as the energy gradients of the IQA energy terms that are most stabilised in forming the transition state must be the opposite of the total system energy gradient. As such, negative REG values imply stabilisation, when going from the reactants to the transition state, whereas positive REG values imply destabilisation. We also stress that the validity of the REG method in this case is evident through the Pearson correlation coefficients *R* in Table 1 all being approximately equal to positive or negative unity.

**Table 1:** The exchange‐correlation IQA energies with largest magnitude REG values are shown, along with their Pearson correlation coefficients *R*. The column marked “Label” is used to mark the curly arrows shown in Figure 6. Note that the prime (i.e. ′) in this and subsequent tables is unrelated to the prime used to distinguish the amino acid residues (e.g. Figure 3).

TERM	Label	REG	R
V_xc_(C58, O66)	A	−4.8	−0.92
V_xc_ (O67, H100)	B	−3.9	−0.97
V_xc_(C35, O36)	C	−2.0	−0.97
V_xc_(O37, H99)	D	−1.1	−0.98
V_xc_(O66, O67)	E	−0.8	−0.99
V_xc_(O11, H101)	F	−0.7	−0.96
V_xc_(O37, O66)	G	−0.5	−0.99
V_xc_(N59, O66)	H	−0.4	−0.97
			
V_xc_(O66, H101)	E′	1.3	0.95
V_xc_(C35, O37)	D′	1.5	0.96
V_xc_(O66, H99)	C′	2.0	0.98
V_xc_(C58, O67)	B′	2.6	0.97
V_xc_(O36, H100)	A′	3.8	0.97

**Figure 6:**
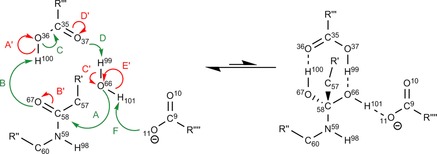
The energetic mechanism as defined by the exchange‐correlation IQA terms with the largest (in terms of absolute value) REG values shown in Table 1. The greens arrows represent bond strengthening/forming and the red ones bond weakening/breaking. For clarity of the diagram, we have left out E, G and H as these are “through‐space” exchange‐correlation interactions (discussed further in the main text). The structure on the right corresponds to the transition state, which explains why the arrow is predominately to the left.

It is possible to represent the results of Table 1 as a mechanism, as shown in Figure 6. The mechanism in Figure 6 differs from a traditional chemical mechanism in its meaning, because it does not directly represent the movement of electrons. Instead, it represents the exchange‐correlation between two topological atoms becoming energetically more favourable or more unfavourable when progressing from IRC sample point 1 to IRC sample point 11. We have assigned colour, direction and labels to the curly arrows to aid in understanding the values given in Table 1. The colour of each arrow represents the sign of the REG term with which it is associated. In Figure 6 green represents a negative REG value and red represents a positive REG value.

Energies with negative REG values regress in the opposite direction compared to the total energy, when going from the reactants to the transition state. The total energy increases in the forward direction (from IRC sample point 1 to 11). Hence, the total energy gradient is positive, because a REG is essentially a ratio of gradients (i.e. partitioned energy over total energy), a negative REG means that the partitioned energy must decrease in the forward direction. Thus, a negative REG value represents stabilisation or bond strengthening (or formation) over the course of the reaction. In contrast, red arrows have positive REG values that represent destabilisation (i.e. regress in the same direction as the total energy) when going from the reactants to transition state. Hence these REG values represent bond weakening (or breaking) over the course of the reaction.

The directionality of the arrows is somewhat arbitrary as the reaction is reversible. However, as stated earlier, we consider the reaction proceeding from IRC Sample Number 1 to 11 in Figure 5 in order to show the mechanism proceeding in the same direction as traditional peptide hydrolysis. To summarise the colour scheme used:

- Red arrows and energy terms both represent the exchange‐correlation energies that become less favourable over the course of the reaction (i.e. indicative of bonds breaking/weakening).
- Green arrows and energy terms both represent the exchange‐correlation energies that become more favourable over the course of the reaction (i.e. indicative of bonds forming/strengthening).

The energetic mechanism determined by the REG values in Table 1 is shown in Figure 6. The first point to note is that the REG‐determined energetic mechanism shows a nucleophilic attack mechanism (which is similar to the traditional mechanism). In fact, the bonds breaking and forming in the traditional mechanism are shown to be of crucial importance as they have the largest magnitude REG values in Table 1 (*A*, *A*′, *B* and *B*′). This shows that the REG method is able to recover well‐known features of reactions by only considering the largest REG values. However, the mechanism determined by the REG method also includes additional curly arrows not included in the traditional mechanism. These additional curly arrows express more subtle effects than those shown in Figure 1. The cascading nature of the arrows in Figure 6, along with the alternating green and red arrows, shows that the REG method is able to show how as every bond strengthens, another weakens. This represents the textbook understanding of valence bonding patterns that only allow for a given number of bonds depending on the number of valence electrons. While a useful “rule of thumb” for the organic chemist, the physical nature of bonding is much subtler and is reflected in our treatment. Indeed, not all the terms found by the REG method are cascading. For example, *E*, *G* and *H* are through‐space bonding changes. Through‐space exchange‐correlation^56^ may not be intuitive to many chemists who are used to integer bond and valence numbers. However, through‐space exchange‐correlation is physically reasonable and is often reflected in textbooks by concepts such as conjugation (which have non‐integer valence numbers) and so‐called “electron deficient” bonding seen often in boron chemistry. Detecting through‐space interactions is one of the benefits of working with IQA over the orbital based method NBO.^33^ This ability to determine through‐space interactions allows for the observation of subtle catalytic effects in the peptide hydrolysis reaction.

The through‐space exchange‐correlation within the substrate mentioned in the previous paragraph is not shown in Figure 6 but present in Table 1. Table 1 shows the increased stability of the through‐space exchange‐correlation between the reactive water oxygen (O66) and the amide oxygen (O67) shown by the energy term labelled *E*. This increased stability could be attributed to partial conjugation of the amide and the reactive water in the transition state. This partial conjugation is also evidenced by energy terms *G* and *H*, which both involve O66 as well. In particular, energy term *G* can be seen as partial conjugation of the aspartic carbonyl oxygen (O37) and the reactive water (O66), while energy term H corresponds to partial conjugation between the amide nitrogen (N59) and the reactive water (O66). Within the IQA method, it is not possible to determine conjugation in the traditional sense because IQA does not explicitly refer to molecular orbitals in its output. However, there is definitely an increase in the exchange‐correlation bonding between the atoms discussed.

To show further how to use the output of the REG method we will discuss a number of REG values shown in Table 1. The REG value with the most negative value in Table 1 (i.e. A) represents the bond formation between O66 and C58, which agrees with the traditional mechanism of bond formation by nucleophilic attack. Indeed, energy terms with more negative REG values show larger relative stability over the course of the reaction, which in turn indicates bond formation compared to mere bond strengthening.

The energy term with the second most negative REG value (B) corresponds to the exchange‐correlation between H100 and O67. This term shows one of the Aspartic groups stabilising the peptide substrate by partially protonating the oxygen of the amide (which starts to form an alcohol). This also shows the asymmetry in the role of the Aspartic groups, such that Asp25 activates the water whereas Asp25(′) not only activates the water but also stabilises the peptide upon nucleophilic attack. The term with the third largest REG value (C) shows the “double” bond forming in the Asp25(′) carbonyl, which can be considered to also facilitate the process discussed previously regarding the curly arrow labelled B. By considering terms *B* and *C* it is possible to see how the residues in the active site facilitate the nucleophilic attack of the water on the peptide. Considering all the other negative REG values in Table 1, it is possible to see in Figure 6 how the active site facilitates peptide hydrolysis in both activating the water molecule and stabilising the peptide in the transition state. It is possible to study a larger subset of exchange‐correlation IQA terms than the one studied here, which would allow for the observation and analysis of more subtle catalytic properties of the active site.

The exchange‐correlation IQA terms with positive REG values (marked in red) destabilise as the reaction progresses and therefore indicate bond‐weakening and bond‐breaking. As indicated by having the most positive REG value, the exchange‐correlation term that most destabilises is that between O36 and H100. This term represents the partial deprotonation of the catalytic Asp25(′), which is required to stabilise the formation of the alcohol group on the substrate during peptide hydrolysis.

The second most positive IQA energy term (B′) determined by the REG method is that of the exchange‐correlation between the amide carbon (C58) and amide oxygen (O67), which represents the weakening of the bond between them. The formally double bond C(58)=O(67) is part of an amide group, together with the formally single bond C(58)−N(59). These bonds are linked via the classical resonance canonicals, which weakens the former and strengthens the latter. Hence, if C(58)=O(67) is weakened, through interaction with an atom external to the amide group, C(58)−N(59) will also be weakened. Thus, the cleaving of the scissile peptide bond is helped by the weakening of C(58)=O(67).

By considering the terms represented by *C*′ and *E*′ we can see the asymmetric activation of the reactive water by the Asp25 and Asp25(′) groups because, traditionally, one hydrogen atom is fully deprotonated by Asp25(′) and the other hydrogen forms a hydrogen bond with the Asp25 carboxyl group. This asymmetry is shown in the REG method by the difference in the two REG values (C′=2.0 and E′=1.3). Finally, we also see bonding rearrangement in the Asp25(′) carboxylic acid group because one of the oxygens (O36) is deprotonated and the other is protonated (O37). As the curly arrows cascade this rearrangement leads to the bond weakening between atoms O66 and H101, as represented by the curly arrow labelled *E*′. The term E′ represents bond weakening (not bond breaking). This can be seen in Table 1, as the REG value for E′ is smaller in magnitude than C′, which indicates less of an energetic change over the course of the reaction. This bond weakening is associated with the hydrogen bond formation between O11 and H101 (term F).

Here we have shown that determining reaction mechanism and catalytic properties within an active sight from REG values is simple. We showed that, by simply looking at ordered lists of REG values, chemical insight can be gained. We can also say that this chemical insight is physically well‐grounded as it is based purely on well‐defined energies.

### Electrostatic contributions to the peptide hydrolysis

In the previous section it was shown how the exchange‐correlation energies could be used to define a reaction mechanism that represents covalent bonds breaking and forming. Although similar to the traditional chemical mechanism, the exchange‐correlation terms do not allow for exhaustive conclusions to be drawn as the electrostatic energy must also be considered. For this reason, we will study the electrostatic terms here. It should be noted that the REG values in Table 2 are directly comparable to those in Table 1.

**Table 2:** The 10 largest REG values (by magnitude) and the associated Pearson correlation coefficient *R* for the electrostatic terms.

TERM	REG	R
V_cl_(O67, H100)	−6.8	−0.97
V_cl_(C35, O36)	−5.6	−0.98
V_cl_(C35, H100)	−3.3	−0.97
V_cl_(C58, O66)	−3.2	−0.98
V_cl_(O37, H99)	−2.9	−0.99
		
V_cl_(C35, O37)	2.4	0.92
V_cl_(C35, H99)	3.0	1.00
V_cl_(O36, H100)	6.5	0.96
V_cl_(C58, N59)	8.1	0.83
V_cl_(C58, O67)	8.8	0.92

Here we discuss some of the largest electrostatic contributions to the overall stability of the reaction mechanism using the REG method. The IQA electrostatic energy that most contributes to the reaction is the electrostatic interaction between the amide oxygen (O67) and the Asp25(′) carboxylic acid hydrogen (H100). The fact that the REG of this electrostatic term is larger than its counterpart exchange‐correlation term (see Table 1) shows that this proton transfer is primarily dominated by electrostatics. Furthermore, we can state that the electrostatic term is primarily polarisation driven. As shown in Table 3, the polarisation REG for this interaction is the most negative and constitutes −6.1/−6.8=90 % of the overall interaction. The two next largest electrostatic REG values relate to the deprotonation of the Asp25(′) group, which was discussed in the previous section. These values show that the electrostatic interactions between the carboxylic carbon (C35) of the Asp25(′) and the carbonyl oxygen (O36), as well as the 1,3 electrostatic interaction between C35 and H100, energetically favour the reaction proceeding. The former favourable electrostatic interaction is likely due to the increased stabilisation as the oxygen charge increases, while the latter is likely due to the increased distance between the positive hydrogen (H100) and the positive carbon (C35) as the hydrogen deprotonates.

**Table 3:** The 13 largest REG values (by magnitude) where the electrostatic potential is split into its two energy contributions: monopolar/charge transfer (CT) and polarisation (PL).

TERM	REG	R
V_PL_(C67, H100)	−6.1	−0.95
V_CT_(C58,O66)	−4.7	−0.99
V_CT_(O66, H101)	−4.2	−0.69
V_PL_(C35, H100)	−3.9	−0.99
V_CT_(C35, O36)	−3.4	−0.99
V_CT_(O66, H100)	−3.2	−0.59
		
V_PL_(C58, O67)	3.4	0.92
V_PL_(O66, H102)	3.4	0.72
V_PL_(O66, H115)	3.7	0.74
V_PL_(O66, H101)	4.7	0.70
V_CT_(C58, N59)	5.2	0.86
V_CT_(C58, O67)	5.4	0.92
V_PL_(O36, H100)	6.9	0.97

The next most negative term represents the electrostatic interaction between the nucleophilic oxygen (O66) of the WR water and the carbon (C58) of the amide (which is attacked). This electrostatic interaction shows that the nucleophilic attack is only partially driven by electrostatics because in the previous section we showed that the attack was also driven by exchange‐correlation. From Table 3, we can see that this nucleophilic attack is primarily based on the monopolar energy, which corresponds to charge transfer and is thus not due to polarisation. We show here that the nucleophilic attack has stabilising contributions from both electrostatic and exchange‐correlation energies. By comparing the REG values of these two energies, we find that both are important as drivers of the reaction, but exchange‐correlation is slightly more of a driving factor as it has a larger REG value, in magnitude, (−4.8, see Table 1) than the electrostatic REG value (−3.2, see Table 2).

Finally, we show that the electrostatic terms that most interact to prevent the interaction are listed with positive values in Table 2. The electrostatic IQA terms that most opposes the reaction are those between the amide oxygen (O67) and the amide carbon (C58), and the amide nitrogen (N59) and the amide carbon (C58). This shows that peptide hydrolysis not only destabilises the exchange‐correlation between the amide atoms (see previous section), but it is also destabilises the amide atoms electrostatically.

Finally, we consider the final energy type, denoted V_inter_(A,B), which is the sum of the corresponding (A,B) exchange‐correlation term and the electrostatic term. The purpose of this final analysis is to allow for any cancellation between the exchange‐correlation and electrostatic pairwise energies, and thereby investigate the total interaction between two atoms. Using the total interaction energy is useful for determining the reaction mechanism because in nature there is no distinction in stability due to exchange‐correlation or electrostatic energy. We can therefore see how the total interaction between two atoms dictates the reaction mechanism. Table 4 reports the results of using the REG method on the total interatomic energies.

**Table 4:** The 11 largest REG values (by magnitude) and the associated Pearson correlation coefficient R for interatomic terms.

TERM	Label	REG	R
Inter_o67_h100	A	−10.8	−0.97
Inter_c58_o66	B	−8.0	−0.97
Inter_c35_o36	C	−7.6	−0.97
Inter_o37_h99	D	−4.0	−0.99
Inter_c35_h100	E	−3.3	−0.97
Inter_o11_h101	F	−2.2	−0.97
			
Inter_c35_h99	E′	2.9	1.00
Inter_c35_o37	D′	4.0	0.94
Inter_c58_n59	C′	8.3	0.83
Inter_o36_h100	B′	10.2	0.97
Inter_c58_o67	A′	11.4	0.94

**Figure 7:**
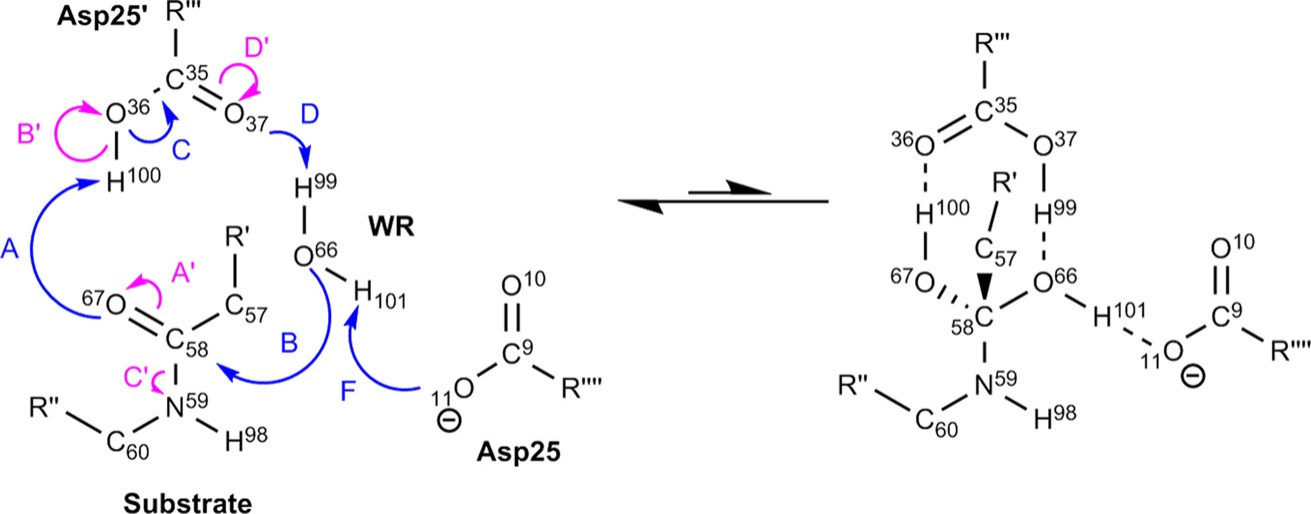
The mechanism drawn from the total IQA interaction energy REG analysis, which links to the labels given in Table 4.

From Table 4 we are able to draw a mechanism much like the one for exchange‐correlation. Figure 7 shows this interatomic energy reaction mechanism. Figure 7 shows many of the same features as in Figure 6, with some notable differences. One such difference is that the hydrogen transfer between the amide oxygen (O67) and the Asp25(′) hydrogen (H100) is more dominant than the nucleophilic attack of the WR oxygen (O66) on the amide carbon (C58). This must be due to the additional electrostatic terms, because the electrostatic REG value (see Table 2) of the former is the most important electrostatic contribution. It is also evident that the deprotonation and bond weakening between the oxygen and the hydrogens in the WR water is not as important as shown in the exchange‐correlation case. Therefore we can state that the WR water is electrostatically bound in the transition state by the Asp25 and Asp25(′) groups. Two very important interactions, shown by Figure 7, that inhibit the reaction occurring are those between the amide carbon (C58) and its neighbouring oxygen (O67) and nitrogen (N59). In particular, the total interaction points to the fact that the destabilisation of the carbon‐nitrogen interaction is a large energy barrier to the reaction occurring.

Pragmatically, we would recommend that using total pairwise IQA interaction energies is the universal method for understanding a reaction mechanism in large systems using the REG method. As it gives the total energy between two atoms and therefore contains the most information about how reactions occur in large systems.

The intra‐atomic energy terms have not yet been considered. In this system we find that the intra‐atomic REG values range from 2.2 to −6.3. This makes the intra‐atomic terms less dominant than the interatomic IQA energy terms, which have a REG value range of 11.4 to −10.8, as seen in Table 4. The intra‐atomic terms are also not as useful in determining reaction mechanism because their purely intra‐atomic nature gives no information regarding atomistic pairwise information.

## Conclusions

In this study of a peptide hydrolysis reaction in the HIV‐1 Protease active site, we have shown that the REG method, when used in conjunction with IQA energies can be used to determine the mechanism of reaction. In using IQA energies we have an energetic scheme that is without arbitrary parameters, well‐defined atoms and without dubious energy terms. The REG method is also parameterless and therefore the determination of the reaction mechanism is achieved here without arbitrary parameters. Furthermore, this reaction mechanism gives much more insight into catalysis than the traditional mechanism and is based on an absolute measure of interatomic energy, not on electron density given the ongoing controversy surrounding the interpretation of bond critical points.

It is pleasing that the REG method reveals “through‐space” interactions. These can be problematic in terms of standard chemical intuition. In this work, however, they roll out naturally and are fully integrated (in terms of computational treatment) with the bonded interactions. This ability to determine through‐space interactions allows for the observation of subtle catalytic effects in the peptide hydrolysis reaction.

This study shows that the REG method is a powerful tool that can be used to automate the analysis of large chemical systems and allows for the exhaustive study of such a system providing chemical insight. The REG method requires no prior knowledge of the system and so can be useful tool in studying protein‐ligand binding, active site reaction mechanisms and conformational changes within large biomolecules. We hope that this work will set an example in how to use IQA in large systems to recover physically meaningful results.

## Theoretical and Computational Background

### Interacting quantum atoms (IQA)

The IQA method was originally derived in its entirety by Blanco et al.^17^ although the electrostatic terms were previously derived and implemented by Popelier and Kosov,^6^ and independently and simultaneously by Salvador^57^ et al. Equation (1) shows the partitioning method used, in which each energetic term is calculated by integrating, in principle, over either the first‐ or second‐order reduced density matrices with the appropriate operator.

(1) $$E_{total}\;=\sum_{A}^{\mathrm{atoms}}E_{\mathrm{intra}}^{A}+\frac{1}{2}\sum_{A}^{\mathrm{atoms}}\sum_{B\neq A}^{\mathrm{atoms}}\left(V_{\mathrm{cl}}^{AB}+V_{\mathrm{xc}}^{AB}\right)=\sum_{A}^{\mathrm{atoms}}E_{\mathrm{intra}}^{A}+\frac{1}{2}\sum_{A}^{\mathrm{atoms}}\sum_{B\neq A}^{\mathrm{atoms}}V_{\mathrm{inter}}^{AB}$$

The first term in Equation (1) requires a 3D integration of the electron density over each atom independently and contains the (one‐electron) intra‐atomic kinetic, exchange‐correlation and electrostatic energies within the atom. The second and third terms are obtained from 6D integrations over two topological atoms *A* and *B*, and represent the (classical) electrostatic and exchange‐correlation energies, respectively. Note that, at long‐range, the electrostatic energy can be calculated^58^ via atomic multipole moments thereby avoiding 6D integration. Because the B3LYP density functional is used in this work, the exchange and correlation energies are combined in Equation (1). The first successful attempt at combining a density functional (in this case B3LYP) with IQA, such that all partitioned energies add up to the system's total energy, was achieved^56^ in 2016. We mention that it is also possible to calculate electron correlation explicitly, again within IQA, for wavefunctions derived from Møller–Plesset perturbation theory^59^, ^60^ and coupled cluster (CCSD(T)).^61^, ^62^

To extract more information from the IQA electrostatic terms, we partition this energy further into monopolar (charge‐transfer)^63^ and polarisation terms, which are defined in Equations (2), (3), respectively.

(2) $$V_{ct}\left(A,B\right)=\frac{Q_{00}^{A}Q_{00}^{B}}{r_{AB}}$$

(3) $$V_{pl}\left(A,B\right)=V_{cl}\left(A,B\right)-V_{ct}\left(A,B\right)$$

in which $Q_{00}^{A}$ is the monopole moment (i.e. essentially atomic net charge) of atom *A* and *r*_AB_ is the internuclear distance between atoms *A* and *B*. These two terms enable further understanding of polarisation and charge transfer without having to invoke any approximations or perturbation theory. The physical picture behind this further partitioning should be clear. First, an atomic monopole moment measures the build‐up or depletion of electronic charge within that atom. For example, the oxygen in water returns a value $Q_{00}^{O}=-1.2$ , expressing the fact that the oxygen has attracted an electronic charge of 0.6 e from each hydrogen. In non‐symmetric cases it is not clear how much charge has been drawn from which other atom but the principle remains that atomic monopole moments express charge transfer. Secondly, we view charge transfer as a special case of polarisation in general. Actually, the term polarisation is most often used in the narrower meaning of dipolar polarisation, which we view as a change in the atomic dipole moment. In other words, and in summary, charge transfer is monopolar polarisation, referring to a change in the zeroth multipole moment, while (dipolar) polarisation refers to a change in the first multipole moment. We briefly mention that polarisation can be predicted by machine learning^64^, ^65^ after suitable training.

### The relative energy gradient (REG) method

We recently published^4^ and applied the REG method to the water dimer as an example of how to understand hydrogen bonding in the system. The water dimer was chosen as a trivial system to illustrate the REG method as a proof of concept. However, the current study is far from trivial because its size is too large to analyse the IQA terms “by hand”.

Here we only repeat the fundamentals of the REG method and recommend the original paper^4^ for more details concerning the logic behind the method. The REG method has two main objectives: (i) to determine subsets of partitioned energies that best represent the total behaviour of the system, and (ii) to extract chemical insight from an energetically partitioned system. Given these two objectives, it is critical that the REG method is exhaustive and does not depend on arbitrary parameters. Central to REG is that the system is systematically perturbed, controlled by a control coordinate *s*. In the current case study, the control coordinate is an intrinsic reaction coordinate (IRC) but it can be a simple internuclear distance, or a dihedral angle. Typically, one needs about a dozen or so snapshots along a relevant trajectory in which the whole system evolves. The REG method then looks for correlations, occurring along such a trajectory, between the total energies and the atomic energies. However, the REG method is applied energy‐barrier‐wise. In other words, the PES is separated into segments that are defined by the turning points in the total energy. We call these segments “barriers” because there is always a direction along the energy profile in which the energy rises towards a local maximum at the turning point, which marks the end of the segment. In the current study there is only one barrier.

The IQA partitioning scheme is additive in nature such that the total energy of the system can be recovered by the following sum,

(4) $$E_{\mathrm{total}}\left(s\right)=\sum_{i=1}^{N}E_{i}\left(s\right)$$

in which *N* is the total number of energy terms and *s* is the control coordinate, which is sampled at *M* data points within a single barrier. Practically speaking, Equation (4) is numerically not exact because of the well known atomic integration error.^66^ However, this error is typically systematic, in that the sum of all IQA energies is typically smaller than the wavefunction's total energy. In this case study, the maximum error found for any of the *M*=11 data points is 26 kJ mol^−1^. Energy gradients are not much affected by this number because of the systematic nature of the error.

The REG method relates the gradient of a partitioned energy (denoted *E*_i_) to the total energy of the system (denoted *E*_total_). It does this using linear regression as shown in Equation (5),

(5) $$E_{i}\left(s\right)=m_{{\mathrm{REG}}_{i}}\cdot E_{total}\left(s\right)+c_{i}$$

in which $m_{REG_{i}}$ is the Relative Energy Gradient, $c_{i}$ is the *y* intercept (which does not contain chemically relevant information) and s is the control coordinate. Note Equation (5) corresponds to an equation for every energy term *i*, and that it is fitted to the *M* data points that represent the barrier. From here onwards, the $m_{REG_{i}}$ value shall be referred to as the *REG* value of a given energy term *i* or simply REG_i_. It is calculated using ordinary least‐squares linear regression as shown in Equation (6),

(6) $$REG_{i}=\frac{(E_{total}^{translated}{)}^{T}\cdot E_{i}^{translated}}{(E_{total}^{translated}{)}^{T}\cdot E_{total}^{translated}}$$

in which *T* denotes the transpose, transforming an *Mx1* column vector into a *1xM* row vector or:

(7) $$\left(E_{i}^{translated}\right){}^{T}=\;\left[E_{i}\left(s_{1}\right)-\bar{E}{}_{i}\;\;\;E_{i}\left(s_{2}\right)-\bar{E}{}_{i}\;\;\cdot \cdot \cdot \;\;E{}_{i}\left(s{}_{M}\right)-\bar{E}{}_{i}\right]{}^{T}\left(E_{total}^{translated}\right){}^{T}=\;[E_{total}\left(s_{1}\right)-\bar{E}{}_{total}\;\;\;E_{total}\left(s_{2}\right)-\bar{E}{}_{total}\;\;\cdot \cdot \cdot \;\;E_{total}\left(s{}_{M}\right)-\bar{E}{}_{total}]{}^{T}$$

The energies in the above equations are translated such that the mean energy is 0, where ${\bar{E}}_{i}$ is the mean of the values in vector $E_{i}$ (and the same is true for the total energy). By definition [see Eq. (5)], a REG value only makes sense if the partitioned energy (*E*_i_) and the total energy correlate linearly, or as linearly as possible. To quantify the degree of linearity we use the Pearson correlation coefficient, which is calculated in Equation (8),

(8) $$R_{i}\;=\frac{\sum_{s}^{M}\left(E_{total}\left(s\right)-{\bar{E}}_{total}\right)\left(E_{i}\left(s\right)-{\bar{E}}_{i}\right)}{\sqrt{\sum_{s}^{M}{\left(E_{total}\left(s\right)-{\bar{E}}_{total}\right)}^{2}}\sqrt{\sum_{s}^{M}{\left(E_{i}\left(s\right)-{\bar{E}}_{i}\right)}^{2}}}=\frac{{\left(E_{total}^{translated}\right)}^{T}\cdot E_{i}^{translated}}{\left|\right|E_{total}^{translated}{\left|\right|}_{2}\left|\right|E_{i}^{translated}{\left|\right|}_{2}}$$

Once the REG values for the system have been calculated, they are ordered from most positive to most negative (i.e. largest to smallest). The magnitude of the REG value gives the ratio of the energy gradient of the partitioned energy compared to the total energy. As such if the REG value has a magnitude of 1 then the partitioned energy has the same magnitude of gradient over the barrier as the total energy. If the REG value magnitude is <1 then the gradient of the partitioned energy is smaller than the total energy, and vice versa. The sign of the REG value gives information on the direction of the gradient: if the sign of the REG value is positive then the partitioned energy gradient acts in the same direction as the total energy over the given barrier (i.e. the partitioned energy behaves in a similar fashion to the total energy). In contrast, if the REG value is negative then the partitioned energy acts in the opposite direction to the total energy over the given barrier (i.e. the partitioned term has the opposite behaviour when compared to the total energy of the system). Being exhaustive, the REG method ranks all IQA energy terms such that the terms with the largest magnitude REG values are more chemically relevant than IQA terms with smaller magnitude REG values. Due to its exhaustive nature, the REG method observes all subtle catalytic effects, and ranks them in a quantitative manner.

## Computational Details

The transition‐state geometry was calculated at the B3LYP/6‐31+G(d,p) level of theory, without geometric constraint on the residues, and had one “negative frequency”, as would be expected for such a stationary point. Although it is not the normal practice to carry out unconstrained optimisations on enzyme fragments, we chose to do so here as the unconstrained structure we determined was not too different from that obtained by Garrec et al.,48a who used constraints. Our approach has, in addition, the advantage of lacking spurious vibrational frequencies causing problems with the determination of the free energy or causing problems for the IRC. Starting from this transition state geometry, an intrinsic reaction coordinate (IRC) calculation was performed for 100 steps. Along the trajectory 11 geometries were selected, including the transition state and the reactants. The wave functions of these 11 geometries were then calculated at B3LYP/6‐31+G(d,p) level. All the density functional calculations were performed using GAUSSIAN09.^67^ The IQA analysis was performed using the program^68^ AIMAll version 16.01.09 (in which the default AIMAll integration method^69^, ^70^ was used instead of the TWOE^71^ algorithm, due to observed instabilities). The REG analysis was performed by the in‐house code ANANKE. The system contains 133 atoms, which means that there are 17 689 individual IQA terms to be calculated. This number can be justified as follows: for an *n*‐atom system there are *n*(*n*‐1)/2 interatomic electrostatic energy terms (V_cl_), *n*(*n*‐1)/2 interatomic exchange‐correlation energy terms (V_xc_), and *n* atomic energy terms. As a result there are 2*n*(*n*‐1)/2*+n*=n^2^ IQA terms, and 133^2^=17 689.

## Conflict of interest

The authors declare no conflict of interest.

## Supporting information

As a service to our authors and readers, this journal provides supporting information supplied by the authors. Such materials are peer reviewed and may be re‐organized for online delivery, but are not copy‐edited or typeset. Technical support issues arising from supporting information (other than missing files) should be addressed to the authors.

Supplementary
