# Supplementary material for: Using the Relative Energy Gradient Method with Interacting Quantum Atoms to Determine the Reaction Mechanism and Catalytic Effects in the Peptide Hydrolysis in HIV‐1 Protease
Source: Chemistry. 2018 Jul 3;24(43):11200–10. doi: 10.1002/chem.201802035 (PMC6099506; doi:10.1002/chem.201802035)
Supplement: Supplementary file 1 — Supplementary [file CHEM-24-11200-s001.pdf]

CHEMISTRY
A European Journal

Supporting Information

Using the Relative Energy Gradient Method with Interacting Quantum Atoms to Determine the Reaction Mechanism and Catalytic Effects in the Peptide Hydrolysis in HIV-1 Protease

Joseph C. R. Thacker, Mark A. Vincent, and Paul L. A. Popelier*[a]

chem_201802035_sm_miscellaneous_information.pdf

Supporting Information

Using the Relative Energy Gradient Method with Interacting Quantum Atoms to Determine the Reaction Mechanism and Catalytic Effects in the Peptide Hydrolysis in HIV-1 Protease

J. C. R. Thacker, M. A. Vincent and P. L. A. Popelier

Table of contents:

Pages 1-4: Cartesian coordinates of the transition state geometry associated with Figure 5 of the main manuscript.

Pages 5-8: Cartesian coordinates of the transition state geometry associated with Figure 5 of the main manuscript.

Transition State Geometry (Ångströms)			
	X	Y	Z
C	5.855875	-0.078562	-6.891524
C	5.545943	0.097353	-5.413764
O	6.342187	0.641016	-4.649643
N	4.325798	-0.364420	-5.005505
C	3.902184	-0.342180	-3.607090
C	4.243395	-1.696449	-2.961076
O	3.664658	-2.732815	-3.314092
C	2.398870	-0.062319	-3.504311
C	1.883238	0.032773	-2.057935
O	2.722010	-0.034907	-1.116521
O	0.630340	0.182723	-1.930545
N	5.207022	-1.687107	-2.011253
C	5.584477	-2.879338	-1.270690
C	4.516728	-3.477549	-0.337463
O	4.693002	-4.612357	0.106094
N	3.428324	-2.713445	-0.071605
C	2.279908	-3.242438	0.639462
C	1.167821	-3.803571	-0.253477
O	0.095847	-4.172545	0.258885
N	1.408954	-3.890812	-1.572239
C	0.420757	-4.413007	-2.501597
C	-9.166209	-1.242430	2.052079
C	-8.901850	0.178430	2.529353
O	-9.621874	0.716188	3.379757
N	-7.840375	0.800673	1.964843
C	-7.469200	2.156472	2.333517
C	6.601344	0.017164	5.987000
C	5.704694	0.035157	4.762531
O	6.114690	-0.341706	3.659907
N	4.431079	0.477503	4.952666
C	3.435220	0.514271	3.874451
C	3.646828	1.786857	3.024385
O	3.000335	2.820884	3.216937
C	2.016442	0.457244	4.437583
C	0.970056	0.295309	3.326692
O	-0.217527	0.640321	3.584733
O	1.383594	-0.195973	2.234540
N	4.608706	1.685467	2.072411
C	5.090584	2.856687	1.352815
C	4.067633	3.572343	0.459420
O	4.234043	4.764186	0.202140
N	3.026719	2.836506	-0.005867

C	1.961860	3.438236	-0.784552
C	0.735681	3.898284	0.011818
O	-0.268745	4.285346	-0.614004
N	0.810507	3.870820	1.348878
C	-0.294795	4.291038	2.198638
C	-7.778213	0.865257	-3.337009
C	-7.807104	-0.643649	-3.530055
O	-8.314024	-1.156256	-4.535468
N	-7.252117	-1.377011	-2.535282
C	-7.235904	-2.828635	-2.586909
C	-3.324960	-4.552693	-0.266797
C	-3.466132	-3.194263	0.392430
O	-4.584789	-2.683754	0.564588
N	-2.308826	-2.597660	0.770160
C	-2.267093	-1.384075	1.561159
C	-1.866164	-0.121511	0.791863
N	-2.560209	0.104122	-0.369156
C	-2.441399	1.367777	-1.081380
C	-3.503924	2.384563	-0.643810
O	-4.684624	2.053532	-0.441510
N	-3.084986	3.658753	-0.573911
C	-3.995387	4.740022	-0.232880
O	-6.011664	-0.331154	-0.074398
O	-0.247692	-0.612571	0.324678
O	-1.706757	0.975147	1.530182
H	5.572753	-0.790537	-1.725680
H	4.465339	0.457229	-3.124497
H	-7.263456	0.315477	1.276278
H	-8.462526	-1.579086	1.286562
H	-9.112200	-1.917226	2.911937
H	-8.311512	2.842010	2.190514
H	-7.169602	2.214153	3.386663
H	-6.634573	2.466887	1.701872
H	-10.184701	-1.295632	1.655760
H	-6.689718	-3.197541	-1.716137
H	-8.252388	-3.239717	-2.577728
H	-6.747125	-3.181165	-3.501922
H	-7.209739	1.180904	-2.458542
H	-7.349018	1.324404	-4.232179
H	-6.852918	-0.919580	-1.713061
H	-8.807596	1.226536	-3.244739
H	-5.419066	0.444057	-0.211670
H	-5.448406	-1.072272	0.224725
H	-2.608025	1.170859	-2.144922

H	-1.563599	-1.508687	2.387912
H	-2.083328	3.865369	-0.614667
H	-3.262959	-1.198855	1.971486
H	-1.427323	-3.090804	0.608719
H	-3.891290	-5.278746	0.324568
H	-3.784812	-4.513256	-1.259001
H	-2.286131	-4.878417	-0.349021
H	-4.314901	4.681942	0.814530
H	-3.481312	5.688667	-0.398561
H	-4.889970	4.695259	-0.860077
H	-1.436693	1.787304	-0.988553
H	-2.729189	-0.721326	-0.924360
H	0.418174	-0.399482	1.076381
H	-1.141268	0.801867	2.375061
H	0.097272	-0.211415	-0.562864
H	2.286727	-3.549058	-1.952429
H	1.657986	3.534765	1.797780
H	0.881638	-4.481566	-3.488224
H	-0.454404	-3.756599	-2.560437
H	-1.171546	3.657399	2.039263
H	0.022207	4.198978	3.237930
H	0.084688	-5.405950	-2.187694
H	-0.567292	5.331131	1.990556
H	1.840889	-2.454661	1.255932
H	2.608396	-4.054594	1.293627
H	2.352166	4.315725	-1.307680
H	1.600179	2.723085	-1.525663
H	3.005543	1.826741	0.120993
H	3.347088	-1.762670	-0.439107
H	5.847909	-3.681501	-1.965075
H	5.451662	3.624508	2.044541
H	6.471322	-2.650589	-0.674082
H	5.931926	2.544707	0.726954
H	6.053769	0.905762	-7.325041
H	6.769298	-0.672259	-6.989114
H	6.938093	-1.009917	6.154433
H	6.112020	0.384235	6.893576
H	5.052202	-0.561682	-7.454633
H	7.487091	0.625780	5.784637
H	3.776559	-0.922472	-5.644235
H	4.172233	0.862106	5.850212
H	1.778363	1.356761	5.012004
H	1.929480	-0.408039	5.106665
H	3.608700	-0.358439	3.242665

H	2.170796	0.879377	-4.016369
H	1.830490	-0.848698	-4.013586
H	5.193446	0.855407	2.111115

Minimum State Geometry (Ångströms)			
	X	Y	Z
C	6.145625	-0.423421	-7.148092
C	5.827321	-0.038396	-5.711275
O	6.440500	0.863343	-5.140852
N	4.816857	-0.743595	-5.124570
C	4.416902	-0.563051	-3.731859
C	4.636969	-1.902885	-3.013567
O	4.199585	-2.958972	-3.488052
C	2.953090	-0.107861	-3.633991
C	2.449998	0.206872	-2.213241
O	3.267327	0.103470	-1.241589
O	1.242611	0.551468	-2.127830
N	5.355414	-1.846241	-1.864202
C	5.606438	-3.023434	-1.053355
C	4.415374	-3.618654	-0.287630
O	4.519893	-4.751945	0.181415
N	3.298965	-2.851016	-0.179516
C	2.085906	-3.367231	0.428759
C	1.029749	-3.865070	-0.560226
O	-0.098536	-4.188438	-0.152123
N	1.388379	-3.962320	-1.853268
C	0.462679	-4.418770	-2.876592
C	-9.287543	-1.229017	2.186469
C	-9.022919	0.205706	2.620082
O	-9.735980	0.765885	3.461502
N	-7.969013	0.814878	2.027506
C	-7.601004	2.183303	2.350423
C	6.702818	0.178091	5.883316
C	5.715349	0.119518	4.732902
O	6.015384	-0.382220	3.647429
N	4.475943	0.639051	4.974086
C	3.422914	0.650474	3.955415
C	3.613867	1.872009	3.024570

O	2.995719	2.925005	3.212820
C	2.041459	0.677537	4.612037
C	0.950816	0.414251	3.585562
O	-0.211146	0.962005	3.891998
O	1.161882	-0.269694	2.590560
N	4.514372	1.702993	2.030692
C	4.982495	2.815533	1.208994
C	3.897545	3.588118	0.440021
O	4.018922	4.804026	0.295712
N	2.856013	2.864340	-0.040561
C	1.767558	3.497627	-0.767022
C	0.580029	3.946907	0.086625
O	-0.457610	4.334872	-0.483822
N	0.710947	3.909798	1.420744
C	-0.352413	4.338956	2.317452
C	-7.760390	0.861579	-3.338640
C	-7.824083	-0.646970	-3.524489
O	-8.309725	-1.152100	-4.543565
N	-7.323502	-1.390327	-2.507679
C	-7.347490	-2.842001	-2.554952
C	-3.496942	-4.359926	-0.547059
C	-3.583398	-3.138648	0.345588
O	-4.679342	-2.686553	0.717229
N	-2.403686	-2.571971	0.700546
C	-2.316108	-1.523977	1.698466
C	-2.291795	-0.085521	1.189647
N	-2.695459	0.139310	-0.068329
C	-2.500850	1.425364	-0.712015
C	-3.635398	2.416703	-0.431367
O	-4.813080	2.048206	-0.284791
N	-3.265103	3.706618	-0.433089
C	-4.232576	4.780581	-0.274151
O	-6.141506	-0.360422	0.003888
O	0.177710	-0.829615	-0.027617
O	-1.962811	0.839481	1.958852
H	5.400055	-0.932350	-1.430771
H	5.072641	0.205241	-3.320651
H	-7.400895	0.312690	1.344689
H	-8.580653	-1.591299	1.435731
H	-9.240348	-1.876307	3.067465
H	-8.437932	2.865843	2.165777
H	-7.322104	2.279000	3.406218
H	-6.753646	2.467356	1.723506
H	-10.303795	-1.292642	1.785808

H	-6.858569	-3.224339	-1.656417
H	-8.374951	-3.221981	-2.597186
H	-6.823206	-3.213135	-3.442598
H	-7.229797	1.167008	-2.433257
H	-7.270917	1.301568	-4.212279
H	-6.941991	-0.940401	-1.673876
H	-8.782023	1.253254	-3.303472
H	-5.548676	0.418413	-0.100944
H	-5.591168	-1.099281	0.330617
H	-2.467037	1.258776	-1.792762
H	-1.398390	-1.662856	2.273204
H	-2.268710	3.944488	-0.455303
H	-3.171014	-1.604351	2.378832
H	-1.529105	-3.004546	0.390599
H	-4.029945	-5.179247	-0.055197
H	-4.019780	-4.149888	-1.485470
H	-2.467938	-4.661269	-0.751805
H	-4.609122	4.835248	0.754222
H	-3.745072	5.724159	-0.526361
H	-5.086677	4.622097	-0.938044
H	-1.537328	1.845954	-0.416720
H	-2.779891	-0.679354	-0.653699
H	0.577003	-0.480800	0.784534
H	-0.897099	0.813854	3.158204
H	0.538117	-0.275943	-0.759326
H	2.320729	-3.679091	-2.132010
H	1.587251	3.597148	1.827533
H	1.005281	-4.504049	-3.819454
H	-0.365422	-3.712616	-3.001611
H	-1.211544	3.663157	2.262536
H	0.037398	4.331691	3.336160
H	0.044751	-5.394197	-2.609474
H	-0.684145	5.350452	2.061876
H	1.611148	-2.589835	1.026735
H	2.352704	-4.208142	1.074688
H	2.138411	4.385866	-1.288042
H	1.379949	2.788071	-1.501057
H	2.933258	1.846562	-0.107226
H	3.296964	-1.885791	-0.514809
H	5.977516	-3.827753	-1.692221
H	5.491001	3.566077	1.821547
H	6.390293	-2.786525	-0.328159
H	5.701898	2.413196	0.489798
H	6.011848	0.457980	-7.782697

H	7.198265	-0.715622	-7.205971
H	7.006599	-0.842965	6.131109
H	6.302728	0.658739	6.780436
H	5.524916	-1.238306	-7.533319
H	7.595513	0.716835	5.553749
H	4.429704	-1.548026	-5.599721
H	4.318729	1.156951	5.827429
H	1.852074	1.635252	5.102943
H	1.984351	-0.117720	5.365589
H	3.530500	-0.265917	3.371795
H	2.827348	0.794350	-4.242666
H	2.291910	-0.868037	-4.066218
H	5.042922	0.836707	2.032700
